# Macrovascular complications in type 2 diabetes: a multiregional study in rural Bangladesh

**DOI:** 10.3389/fendo.2026.1724957

**Published:** 2026-01-26

**Authors:** Bodrun Naher Siddiquea, Dianna J. Magliano, Maima Matin, Afsana Afroz, Baki Billah

**Affiliations:** 1 Department of Epidemiology and Preventive Medicine, School of Public Health and Preventive Medicine, Monash University, Melbourne, VIC, Australia; 2 Prothikrit Institute of Health Studies (PIHS), Dhaka, Bangladesh; 3 Diabetes and Population Health, School of Public Health and Preventive Medicine, Monash University, Melbourne, VIC, Australia; 4 Baker Heart and Diabetes Institute, Melbourne, VIC, Australia; 5 Institute of Genetics and Animal Biotechnology of the Polish Academy of Sciences, Jastrzebiec, Poland; 6 Obstetrics and Gynaecology, Faculty of medicine, Dentistry and health Sciences, The University of Melbourne, Melbourne, VIC, Australia

**Keywords:** Bangladesh, coronary artery disease, diabetic foot, machine learning algorithm, macrovascular complications, stroke, Type 2 diabetes

## Abstract

**Objectives:**

To assess the prevalence and determinants of macrovascular complications (coronary artery disease, stroke, and diabetic foot) among adults living with T2DM in rural Bangladesh.

**Methods:**

A population-based cross-sectional study was conducted between December 2023 and September 2024, involving 1094 adults with diagnosed T2DM from rural areas of three regions/divisions in Bangladesh. Data were collected through household interviews, physical examination, and medical record reviews. Macrovascular complications were identified using clinical criteria and documented diagnosis. The leverage of six machine learning (ML) algorithms were applied in identifying influential variables associated with these complications.

**Results:**

The prevalence of coronary artery disease (CAD), stroke, and diabetic foot was 11.2%, 5.3%, and 9.1%, respectively. The Light Gradient Boosting Machine algorithm performed best for CAD and diabetic foot, with ROC values of 98.8% and 92.6%, respectively, while Random Forest showed the best performance for stroke with a ROC of 99%. These models also outperformed others across accuracy, precision, F1 score, and calibration. Across models, common predictors included older age, longer diabetes duration, diabetes onset at age 45 years or above, and smoking. Hypertension and elevated cholesterol were linked to CAD and stroke. Coexisting microvascular complications were also identified.

**Conclusions:**

This study identified a substantial burden of macrovascular complications among rural adults with T2DM, with CAD, stroke, and diabetic foot emerging as the most prevalent outcomes. Advanced age, longer duration of diabetes, smoking, hypertension, and elevated cholesterol were consistently associated with these complications, highlighting the need for intensified cardiometabolic risk control within primary care. These findings underscore the urgency of strengthening integrated diabetes–cardiovascular management in rural Bangladesh to reduce the progression and impact of these major vascular outcomes.

## 1 Introduction

Type 2 diabetes mellitus (T2DM) is an escalating global health concern, particularly in low- and middle-income countries (LMICs), where majority of new cases are emerging (1). In 2024, an estimated 589 million adults were living with diabetes globally, and this number is projected to rise to 853 million by 2050 (1). This increase is especially pronounced in developing countries, driven by rapid urbanization and lifestyle changes (2). Bangladesh, a developing country in the South-East Asia region, bears one of the highest burdens, with a national adult (aged 20–79 years) diabetes prevalence of 13.2% reported in 2024 (1). One of the most serious consequences of T2DM is the development of macrovascular complications, which significantly contribute to morbidity, mortality, and healthcare costs (3).

Macrovascular complications in people with T2DM result from insulin resistance and chronic hyperglycaemia, which damage large blood vessels. These complications include coronary artery disease (CAD), stroke, and peripheral vascular disease (PVD)—often presenting as diabetic foot (4). Individuals with diabetes are two to four times more likely to develop cardiovascular disease than the general population and have a two to five times greater risk of dying from these conditions (5). Cardiovascular complications affect up to 80% of people with T2DM and are responsible for approximately 65% of deaths in this population (6, 7).

While macrovascular complications are common, their prevalence varies across populations. Globally, approximately 12.7% of people with T2DM experience macrovascular complications, with the highest rates observed in South-East Asia (26.7%) and the lowest in Europe (4.0%) (8). Specific complications include CAD (8.2%), heart failure (3.3%), stroke (2.2%), and PVD (1.2%). In LMICs, reported prevalence rates vary widely ranging from 1–40% for PVD, 5–10% for myocardial infarction, and 1–27% for ischemic heart disease (8). In Bangladesh, studies have reported a varied prevalence of macrovascular complications: 25.8% to 31.8% for CAD, 10.1% to 12.7% for stroke, and 12% to 13.4% for diabetic foot (9, 10). Although population-based data on macrovascular complications remain limited, hospital-based and clinical studies suggests a substantial burden. A multicentre study of adults with T2DM reported CAD in approximately 30.5% of participants, stroke in 10.1%, and diabetic foot in 12.0% of participants (9). Similarly, a tertiary-care hospital study found that over half of individuals with diabetes had at least one macrovascular complication, with CAD present in 31.8% and stroke in 12.7% (10). Another hospital-based study documented CAD prevalence of 25.8%, stroke at 11%, and PVD at 14% among individuals with T2DM (11). These findings, while not population-based, highlight a considerable burden of macrovascular complications among people with diabetes in Bangladesh and underscore the need for community-level evidence.

In contrast to high-income countries (HICs), where improved glycaemic control and early intervention have reduced diabetes-related complications, LMICs like Bangladesh face persistent challenges. Delayed diagnosis, limited access to healthcare, and poor adherence to treatment continue to impede effective prevention and management (12). People with T2DM in rural areas are particularly vulnerable due to low health literacy, inadequate screening, and limited access to specialist care (13).

Although some data exist from hospital-based or mostly urban studies, there is limited evidence on the burden and determinants of macrovascular complications among people with T2DM in rural settings. Understanding the prevalence and associated risk factors in these communities is essential for informing integrated and locally appropriate strategies for prevention and care. Previous studies have primarily relied on classical regression methods to identify associated factors. In contrast, machine learning (ML) algorithms are increasingly recognised in public health for their ability to uncover complex risk factor patterns and improve outcome prediction. Unlike traditional approaches, ML can analyse large and diverse datasets, capture nonlinear relationships, and offers considerable promise for strengthening health outcomes, particularly in resource-constrained settings. Therefore, this study aimed to determine the prevalence of major macrovascular complications such as CAD, stroke, and diabetic foot among adults with T2DM living in rural Bangladesh, and to identify the key socio-demographic, clinical, and behavioural factors associated with these outcomes.

## 2 Methods

### 2.1 Study design and setting

This population-based cross-sectional study was conducted in rural Bangladesh between December 2023 and September 2024, as part of a broader research initiative on glycaemic control, major complications, and comorbidities among individuals with T2DM. Data were collected from villages within three union parishads, the lowest administrative units in rural Bangladesh, across three divisions (the highest administrative regions). The selection of divisions was guided by diabetes prevalence: Dhaka division, with the highest reported prevalence (15%), was purposively selected, while Rajshahi and Khulna divisions were randomly chosen from those with moderate prevalence levels (ranging from 5.6% to 10.9%) (14). In each division, one district was selected, followed by one upazila (subdistrict) from that district. From each upazila, one union parishad was randomly chosen, and the villages within these union parishads served as the data collection sites.

### 2.2 Sample size and participants

The sample size was calculated based on a study that assessed glycaemic control using haemoglobin A1c (HbA1c) levels (15). Using a 95% confidence level, 5% significance level, 2.5% margin of error, and an estimated 70% prevalence of inadequate glycaemic control from a previous study in Bangladesh (15), the required sample size was determined to be 1098 participants. This same sample was also used to assess the prevalence of diabetes-related complications. Finally, 1094 adults (aged ≥18 years) with diagnosed T2DM were recruited for the study. This sample size also maintains similar or better power for macrovascular complications.

### 2.3 Data collection

The data collection process comprised three key components: a structured questionnaire, physical examination, and medical record review. A semi-structured, interviewer-administered questionnaire in English was initially developed based on existing literature and validated instruments, with input from a multidisciplinary team including epidemiologists and medical professionals. To ensure linguistic and conceptual accuracy, the questionnaire was translated into Bangla and then back-translated into English by bilingual experts. A panel of reviewers assessed all versions to establish semantic and construct validity. The questionnaire was pre-tested among 24 eligible participants to evaluate its clarity, feasibility, and reliability. The pilot results demonstrated high internal consistency, with an overall Cronbach’s alpha of 0.93 (**Supplementary Material: Appendix 1**).

Community engagement played a vital role in participant recruitment. Local influencers including teachers, religious leaders, elders, and community representatives, were consulted to support awareness and foster trust. Informational flyers outlining the study objectives were distributed at community hubs such as shops, religious institutions, and schools. Word-of-mouth referrals further facilitated outreach. A trained research team consisting of a diabetes nurse, field supervisor, and local assistants conducted household visits, starting from the periphery of each village. Prospective participants were informed about the study, and written informed consent was obtained from eligible individuals (99.6%) after providing them with an explanatory statement.

Interviews were conducted to collect information on socio-demographics, diabetes-related history, lifestyle behaviours, comorbidities, and complications. Medical records were reviewed using a checklist to extract information on blood tests, diagnosed diseases, and medications. Standardised procedures were followed to measure anthropometric measurements, including height, weight, and waist and hip circumference, as well as blood pressure (BP).

### 2.4 Outcome variables

Complications were identified through a combination of medical record review, clinical assessment, and patient self-reporting. CAD was determined based on documented diagnoses, a history of relevant medical procedures, or the use of medications prescribed for CAD management. A history of stroke was established either through medical documentation indicating a prior cerebrovascular accident or self-reported events consistent with stroke symptoms in the past. Diabetic foot was assessed through direct visual inspection for foot ulcers, deformities, or amputations, in addition to any documented diagnosis in the medical record.

### 2.5 Explanatory variables

The explanatory variables included socio-demographic characteristics (age, gender, education, occupation, marital status, individual and family income, and family size), lifestyle and behavioural factors (history of smoking and smokeless tobacco, physical activity), and clinical characteristics (duration of diabetes, age at onset of diabetes, type of diabetes treatment, list of medications, glycaemic control, body mass index, waist-hip ratio, and history of hypertension). Comorbidities and complications such as dyslipidaemia, retinopathy, neuropathy, and nephropathy were also recorded from medical records.

### 2.6 Operational definitions

Glycaemic control was assessed using HbA1c levels, categorised as adequate (<7%) or inadequate (≥7%) (16). Physical activity was assessed using a modified version of the Global Physical Activity Questionnaire (GPAQ) (17). Diabetes-related distress was measured using the 17-item Diabetes Distress Scale (DDS17/BDDS17) (18, 19). Body Mass Index (BMI) was classified according to WHO guidelines for the Asian population: underweight (≤18.50 kg/m²), normal (18.50–22.99 kg/m²), overweight (23.00–27.49 kg/m²), and obese (≥27.50 kg/m²) (20). WHR was evaluated using cut-off points specific to the Asian population: 85 cm for men and 75–85 cm for women (21). Hypertension was defined as systolic BP ≥140 mmHg and/or diastolic BP ≥90 mmHg, a prior medical diagnosis, or current use of antihypertensive medication. Dyslipidaemia was defined as either a documented diagnosis or the use of lipid-lowering medications. Neuropathy was identified through a documented diagnosis or a score of 7 or more on the Michigan Neuropathy Screening Instrument (22).

### 2.7 Statistical analysis

Data collection, storage, and management were carried out using the Research Electronic Data Capture (REDCap) software (23). Continuous variables were summarised as means with standard deviations (SD) or medians with interquartile ranges, while categorical variables were described using frequencies and percentages. A two-step approach was employed for variable selection, integrating both conventional statistical techniques and machine learning (ML) methods. Initially, univariate analyses were conducted using the chi-square test to assess associations between the outcome variable and potential predictors. Simultaneously, the Boruta algorithm, a machine learning technique based on the random forest classifier, was applied to evaluate the relative importance of variables (**Supplementary Figure S1**). This combined strategy enhanced the robustness of variable selection by leveraging the complementary strengths of traditional statistics and ML.

Based on existing literature, following six ML algorithms were selected: Extra Trees Classifier (ETC), Gradient Boosting (GB), Light Gradient Boosting Machine (LGB), Random Forest (RF), and Support Vector Machine (SVM) and logistic regression (LR) (24–26). All statistical analyses were conducted using Stata version 17, R version 4.4.1, and Python (via the Anaconda platform). A p-value of ≤0.05 was considered statistically significant.

#### 2.7.1 Predictive variable selection and model evaluation

Variable selection is a critical step in enhancing model performance and simplifying analysis by identifying the most relevant variables within a dataset. To determine the most influential variables, SHapley Additive exPlanations (SHAP) were employed (27).

Model optimisation was achieved through hyperparameter tuning combined with 5-fold cross-validation. In this approach, the dataset was divided into five equal subsets; during each iteration, four subsets (80%) were used for training the model and one subset (20%) for testing. Performance metrics, including the area under the receiver operating characteristic (ROC) curve, accuracy, sensitivity, specificity, precision, recall, F1 score, Brier score, and calibration, were computed and averaged across all folds. The algorithm demonstrating the best overall performance was selected as the final predictive model.

#### 2.7.2 Model parsimony

Parsimony in the optimal ML algorithm was achieved by balancing model simplicity with predictive performance, as measured by the area under the ROC curve. SHAP values were used to rank variable importance, and the least influential variable was sequentially removed in an iterative process. After each exclusion, the model was recalibrated and reassessed. This process continued until an optimal subset of variables was identified, minimising the number of variables while maintaining high discriminative performance.

#### 2.7.3 Risk quantification

ML algorithm uses nonparametric approach to identify the most influential variables that enhance the model’s predictive accuracy. Hence, it neither quantifies the risk associated with a variable nor relies on statistical significance. To complement this, the top influential variables selected by ML algorithm can be added to classical LR model to quantify the risk.

### 2.8 Ethics approval

Written informed consent was obtained from all participants, with thumbprints used for those who were illiterate. Ethical approval was granted by the Monash University Human Research Ethics Committee (MUHREC, Project No. 39979) and the Ethical Review Committee of the Bangladesh University of Health Sciences (Memo No. BUHS/ERC/EA/23/54).

## 3 Results

### 3.1 Sociodemographic and life-style behaviours

A total of 1094 individuals with T2DM were included in the study, of whom 72.6% were women. The mean age of participants was 51.4 ± 12.1 years. Most participants (94.1%) had an education level below secondary school, and approximately 69% were housewives (**Table 1**). The average duration of diabetes was 6.8 ± 6.1 years.

**Table 1:** Sociodemographic characteristics and life-style behaviours of the study participants.

Variables	Total	CAD^**^		Stroke		Diabetic foot	
	N = 1094	n = 123		n = 58		n = 100	
	n (%)	n (%)	p-value^*^	n (%)	p-value^*^	n (%)	p-value^*^
Socio-demographics							
Gender							
Male	300 (27.4)	40 (13.3)	0.179	25 (8.3)	**0.006**	23 (7.7)	
Female	794 (72.6)	83 (10.5)		33 (4.2)		77 (9.7)	0.928
Age group (years)							
18-39	167 (15.3)	7 (4.2)		2 (1.2)		6 (3.6)	
40-59	614 (56.1)	67 (10.9)		30 (4.9)		61 (9.9)	
≥60	313 (28.6)	49 (15.7)	**0.001**	26 (8.3)	**0.003**	33 (10.5)	**0.025**
Current marital status							
Unmarried	150 (13.7)	19 (12.7)		6 (4.0)		13 (8.7)	
Married	944 (86.3)	104 (11.0)	0.552	52 (5.5)	0.444	87 (9.2)	0.828
Education level							
Up to secondary school	1029 (94.1)	115 (11.2)	0.779	53 (5.1)		97 (9.4)	0.192
Above secondary school	65 (5.9)	8 (12.3)		5 (7.7)	0.375	3 (4.6)	
Employment status							
Employed	257 (23.5)	30 (11.7)		14 (5.4)		17 (6.6)	
Housewives	758 (69.3)	77 (10.2)		31 (4.1)		75 (9.9)	
Retired	79 (7.2)	16 (20.2)	**0.025**	13 (16.5)	**<0.001**	8 (10.1)	0.275
Family size							
Small (≤4 people)	636 (58.1)	75 (11.8)	0.498	30 (4.7)		60 (9.4)	0.692
Large (>4 people)	458 (41.9)	48 (10.5)		28 (6.1)	0.309	40 (8.7)	
Family income (BDT)							
<=20000 TK	900 (82.3)	102 (11.3)	0.839	49 (5.4)		85 (9.4)	
>20000 TK	194 (17.7)	21 (10.8)		9 (4.6)	0.650	15 (7.7)	0.453
Lifestyle factor							
Smoking							
Never smoked	966 (88.3)	96 (9.9)		45 (4.7)		86 (8.9)	
Past/current smoker	128 (11.7)	27 (21.1)	**<0.001**	13 (10.2)	**0.009**	14 (10.9)	0.453
Smokeless tobacco							
Never used	775 (70.8)	79 (10.2)		40 (5.2)		73 (9.4)	
Past/current user	319 (29.2)	44 (13.8)	**0.087**	18 (5.6)	0.747	27 (8.5)	0.618
Physical activity							
Active	307 (28.1)	39 (12.7)	0.340	14 (4.6)		25 (8.1)	
Inactive	787 (71.9)	84 (10.7)		44 (5.6)	0.494	75 (9.5)	0.475

*p-value representing univariate association between exposures and complications. Bold values are statistically significant (p < 0.05); CAD, Coronary artery disease; Physical activity: active: >150 minutes of walking per week.

### 3.2 Clinical characteristics

A family history of diabetes and hypertension was reported by 36.5% and 32.6% of participants, respectively. Dyslipidaemia was present in 13.5% of the sample. Hypertension (defined as BP ≥140/90 mmHg) was found in 62.8% of participants. Regarding diabetes treatment, 50.9% were on a single oral antidiabetic drug (OAD), 31.1% were on a combination of OADs, and 16.8% were using either a combination of OADs and insulin or insulin alone (**Table 2**). None of the participants used insulin pumps or continuous glucose monitoring devices.

**Table 2:** Clinical characteristics of the study participants.

Variables	Total	CAD^**^		Stroke		Diabetic foot	
	N = 1094	n = 123		n = 58		n = 100	
	n (%)	n (%)	p-value^*^	n (%)	p-value^*^	n (%)	p-value^*^
Clinical characteristics							
Diabetes duration group							
≤5 years	461 (42.1)	46 (10.0)		21 (4.6)		37 (8.0)	
6–10 years	440 (40.2)	45 (10.2)		19 (4.3)		41 (9.3)	
>10 years	193 (17.7)	32 (16.6)	**0.035**	18 (9.3)	**0.023**	22 (11.4)	0.388
Age of DM onset							
≤45 years	617 (56.4)	54 (8.8)		28 (4.5)		55 (8.9)	
>45 years	477 (43.6)	69 (14.5)	**0.003**	30 (6.3)	0.200	45 (9.4)	0.767
Diabetes treatment							
One oral medicine	574 (50.9)	57 (9.9)		30 (5.2)		55 (9.6)	
More than one oral medicine	335 (31.1)	34 (10.1)		12 (3.6)		22 (6.6)	
Insulin only	77 (7.0)	13 (16.9)		5 (6.5)		8 (10.4)	
Oral medicine + insulin	107 (9.8)	19 (17.8)	**0.039**	11 (10.3)	**0.058**	15 (14.0)	0.111
Glycaemic control							
Adequate	256 (23.4)	38 (14.8)	**0.037**	18 (7.0)	0.158	17 (6.6)	
Inadequate	838 (76.6)	85 (10.1)		40 (4.8)		83 (9.9)	0.113
Dyslipidaemia							
No	946 (86.5)	57 (6.0)		20 (2.1)		76 (8.0)	
Yes	148 (13.5)	66 (44.6)	**<0.001**	38 (25.7)	**<0.001**	24 (16.2)	**0.001**
Body Mass Index							
Underweight/Normal	432 (39.5)	38 (8.8)		27 (6.3)		48 (11.1)	
Overweight	449 (41.0)	58 (12.9)		23 (5.1)		40 (8.9)	
Obese	213 (19.5)	27 (12.7)	0.117	8 (3.8)	0.403	12 (5.6)	0.074
Waist-hip ratio							
Low	61 (5.6)	7 (11.5)		6 (9.8)		11 (18.0)	**0.008**
Moderate	153 (14.0)	17 (11.1)		7 (4.6)		7 (4.6)	
High	880 (80.4)	99 (11.2)	0.997	45 (5.1)	0.257	82 (9.3)	
Hypertension							
No	407 (37.2)	14 (3.4)		8 (2.0)		23 (5.6)	
Yes	687 (62.8)	109 (15.9)	**<0.001**	50 (7.3)	**<0.001**	77 (11.2)	**0.002**
CAD							
No	971 (88.8)	–		15 (1.5)		78 (8.0)	
Yes	123 (11.2)	–		43 (35.0)	**<0.001**	22 (17.9)	**<0.001**
Stroke							
No	1036 (94.7)	80 (7.7)		–		88 (8.5)	
Yes	58 (5.3)	43 (74.1)	**<0.001**	–		12 (20.7)	**0.002**
Diabetic foot							
No	994 (90.9)	101 (10.2)		46 (4.6)		–	
Yes	100 (9.1)	22 (22.0)	**<0.001**	12 (12.0)	**<0.001**	–	
Retinopathy							
No	968 (88.5)	76 (7.9)		35 (3.6)		65 (6.7)	
Yes	126 (11.5)	47 (37.3)	**<0.001**	23 (18.2)	**<0.001**	35 (27.8)	**<0.001**
Nephropathy							
No	1062 (97.1)	108 (10.2)		58 (4.7)		86 (8.1)	
Yes	32 (2.9)	15 (46.9)	**<0.001**	8 (25.0)	**<0.001**	14 (43.8)	**<0.001**
Neuropathy							
No	897 (82.0)	85 (9.5)		37 (4.1)		61 (6.8)	
Yes	197 (18.0)	38 (19.3)	**<0.001**	21 (10.7)	**<0.001**	39 (19.8)	**<0.001**
Diabetes distress							
Little or no distress	331 (30.3)	36 (10.9)		18 (5.4)		51 (15.4)	**<0.001**
Moderate distress	394 (36.0)	32 (8.1)		10 (2.5)		20 (5.1)	
High distress	369 (33.7)	55 (14.9)	**0.012**	30 (8.1)	**0.003**	29 (7.9)	

*p-value representing univariate association between exposures and complications. Bold values are statistically significant (p < 0.05); CAD, Coronary artery disease; DM, Diabetes mellitus; ^**^Glycaemic control: adequate: HbA1c <7%, inadequate: HbA1c ≥7%; ^¥^BMI, body mass index (kg/m^2^): underweight: ≤18.50, normal: 18.50–22.99, overweight: 23.00–27.49, and obese: ≥ 27.50; Waist-hip ratio: low: ≤90 cm for men and ≤80 cm for women; moderate: >90 to ≤95 cm for men, >80 to ≤85 cm for women; high: >95 cm for men, >85 cm for women; **^⁋^**Hypertension: present: systolic blood pressure ≥140 mmHg and/or diastolic blood pressure ≥90 mmHg; **^‡^**Diabetes distress: present: ≥2.

### 3.3 Prevalence of macrovascular complications

The prevalence of CAD, stroke, and diabetic foot was 11.2%, 5.3%, and 9.1%, respectively. All three complications were more prevalent among individuals aged ≥60 years and those with comorbid conditions such as hypertension, dyslipidaemia, retinopathy, nephropathy, and neuropathy. CAD was also more common among those with a history of smoking, a diabetes duration of ≥10 years, those with adequate glycaemic control and those using both OAD and insulin, and individuals experiencing high diabetes distress. Stroke was significantly more prevalent among males, individuals with a history of smoking and those on combined OAD and insulin therapy, and those reporting high diabetes distress. Diabetic foot was notably associated with low waist-hip ratio and high levels of diabetes distress (**Table 2**).

### 3.4 Performance metrics of ML algorithms

**Table 3** shows the performance metrics of six ML algorithms. Based on ROC and other performance metrics, the best performing models were LGBM for CAD and diabetic foot, and RF for stroke. The ROC values of LGBM for CAD and diabetic foot were 96.2% (95% CI: 92.4%–99.9%) and 92.6% (95% CI: 86.8%–98.2%), respectively, and that of RF for stroke was 98.5% (95% CI: 97.2%–99.9%). These models also showed improved calibration which was measured by calibration curve (**Supplementary Figure S2**) and brier score.

**Table 3:** Prediction performance metrics of various machine learning algorithms for CAD, stroke and diabetic foot.

Outcome	MLA^*^	Measurement metrics								
		ROC	CI	Accuracy	Sensitivity	Specificity	Precision	Recall	F1-score	Brier score
CAD	RF	0.939	0.891-0.985	0.93	0.36	0.99	0.93	0.68	0.74	0.07
	GBC	0.500	0.414-0.585	0.89	0.00	1.00	0.44	0.50	0.47	0.11
	ETC	0.911	0.855-0.966	0.92	0.32	0.99	0.94	0.66	0.72	0.08
	SVM	0.861	0.793-0.927	0.91	0.35	0.98	0.83	0.67	0.71	0.09
	LGBM	**0.962**	0.924-0.999	**0.94**	**0.63**	0.99	0.91	**0.81**	**0.85**	**0.06**
	LR	0.883	0.820-0.945	0.91	0.34	0.98	0.83	0.66	0.71	0.09
Stroke	RF	**0.985**	0.972-0.999	**0.99**	**0.98**	**1.00**	**1.00**	**0.99**	**1.00**	**0.001**
	GBC	0.500	0.378-0.621	0.95	0.00	1.00	0.47	0.50	0.49	0.053
	ETC	0.953	0.891-0.999	0.95	0.16	0.99	0.93	0.58	0.62	0.045
	SVM	0.914	0.834-0.993	0.96	0.16	0.99	0.93	0.58	0.62	0.043
	LGBM	0.980	0.961-1.010	0.98	0.79	0.99	0.97	0.90	0.93	0.016
	LR	0.936	0.867-0.999	0.96	0.43	0.98	0.77	0.71	0.74	0.040
Diabetic foot	RF	0.880	0.810-0.949	0.91	0.04	1.00	0.96	0.52	0.52	0.087
	GBC	0.500	0.406-0.593	0.91	0.00	1.00	0.45	0.50	0.48	0.091
	ETC	0.852	0.775-0.927	0.91	0.05	1.00	0.96	0.53	0.52	0.086
	SVM	0.792	0.706-0.876	0.91	0.02	1.00	0.96	0.51	0.50	0.091
	LGBM	**0.926**	0.868-0.982	**0.93**	**0.43**	0.99	0.85	**0.71**	**0.76**	**0.068**
	LR	0.602	0.505-0.698	0.91	0.00	1.00	0.45	0.50	0.48	0.091

^*^MLA, Machine learning algorithm; ROC, receiver operating characteristics; CI, confidence interval; CAD: Coronary artery disease; RF: Random Forest; GBC: Gradient Boosting Classifier; ETC: Extra Trees Classifier; SVM: Support Vector Machine; LGBM: Light Gradient Boosting Machine; LR: Logistic regression. Higher metric values indicate better prediction. F1 score balances precision and recall, with higher values reflecting stronger performance. Brier score measures the mean squared difference between predicted probabilities and actual outcomes, where lower values indicate better accuracy.

### 3.5 Predictive variables in the best-performing algorithms

**Figure 1:**
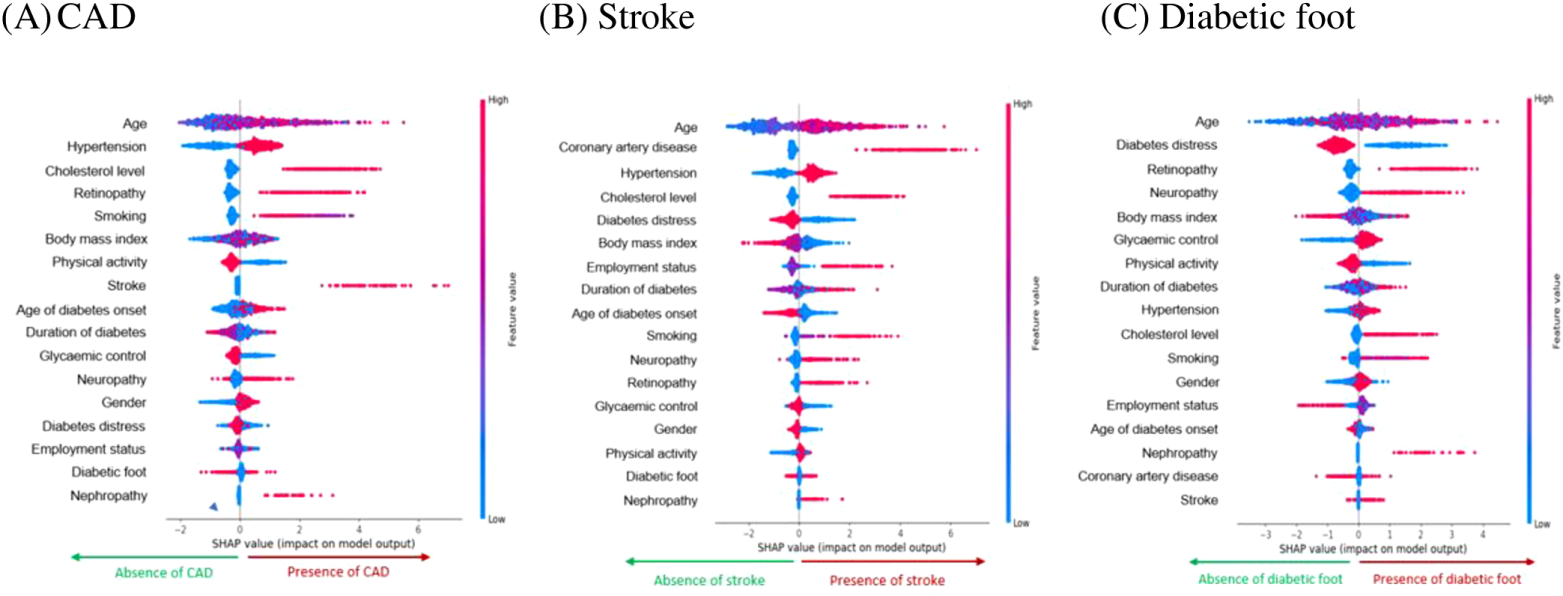
Most influential variables ranked from highest to lowest by LGBM and RF models for **(A)** CAD, **(B)** Stroke, and **(C)** Diabetic foot. LGBM, Light Gradient Boosting Machine; RF, Random Forest; CAD, Coronary artery disease.

**Figure 1** presents a Beeswarm plot, which offers a compact visual summary of how different variables influence the model’s predictions. Variables are ranked by importance, with those at the top (e.g. age) having the greatest impact. The horizontal axis displays SHAP values, where positive values indicate a higher likelihood of complication and negative values suggest its absence. Each dot represents an individual participant, with red indicating higher variable values and blue representing lower values. For numerical variables, the colour gradient reflects actual measurement values, while for categorical variables, it denotes assigned statistical codes.

According to this plot, the top 10 variables associated with CAD are age, hypertension, cholesterol level, retinopathy, smoking, BMI, physical activity, stroke, age of diabetes onset, and duration of diabetes. For stroke, the most important predictors include age, CAD, hypertension, cholesterol level, diabetes distress, BMI, employment status, duration of diabetes, age of diabetes onset, and smoking. The key variables linked to diabetes foot are age, diabetes distress, retinopathy, neuropathy, BMI, glycaemic control, physical activity, duration of diabetes, hypertension, and cholesterol level.

The plot highlighted age as the most influential variable across all three macrovascular complications, with older individuals more likely to experience these outcomes. Additionally, a longer duration of diabetes and onset of diabetes at an older age were predictors of all three complications. Hypertension and high cholesterol levels were consistently associated with increased risk of CAD and stroke. The presence of retinopathy was common predictor across CAD, stroke and diabetic foot, while neuropathy specifically contributed to diabetic foot. Smoking was strongly linked to both CAD and stroke, and also emerged as a key variable for diabetic foot. Lower levels of diabetes distress and lower BMI were notably associated with both stroke and diabetic foot, whereas higher BMI was related to CAD.

In addition to ranking predictors, the plot illustrates the direction and variability of effects, with wider SHAP value distributions indicating substantial inter-individual heterogeneity. Higher levels of hypertension, cholesterol, smoking, older age and longer diabetes duration were consistently associated with increased risk, whereas greater lower BMI showed protective effects across models.

### 3.6 Risk quantification of key influential predictive variables

To quantify the risk of macrovascular complications associated with variables identified in the ML algorithms, top 10 variables related to each complication were applied to a multiple LR model (**Supplementary Table S1**). For example, individuals with hypertension and high cholesterol levels had 2.9-fold (95% CI: 1.48–5.81) and 4.9-fold (95% CI: 2.90–8.40) higher odds of CAD, respectively. Additionally, being overweight and obese was associated with 75% (95% CI: 1.01–3.06) and 54% (95% CI: 0.78–3.04) increased odds of developing CAD. Similar to CAD, high cholesterol levels had 9.2-fold (95% CI: 4.06–21.07) higher odds of stroke. Individuals with retinopathy and neuropathy had 3.9-fold (95% CI: 2.32–6.46) and 3.7-fold (95% CI: 2.25–6.19) higher odds of diabetic foot, respectively.

## 4 Discussion

This study estimated the prevalence of macrovascular complications and applied ML algorithms to identify factors associated with these conditions among individuals with T2DM in rural Bangladesh. A considerable burden of disease was observed, with CAD, stroke, and diabetic foot present in 11.2%, 5.3%, and 9.1% of participants, respectively. In terms of predictive modelling, this study demonstrated that ML algorithms offer valuable insights into the determinants of macrovascular complications. Across the best-performing models (LGBM and RF), older age, longer diabetes duration, hypertension, elevated cholesterol, smoking, physical inactivity, and diabetes-related distress appeared as influential predictors for each complication.

Previous studies have highlighted important rural–urban differences in the prevalence of diabetes and its complications in South Asia, including Bangladesh. A nationally representative study reported diabetes prevalence of 10.8% in urban areas compared to 7.4% in rural populations, consistent with broader regional patterns where urban-to-rural prevalence ratios range from 1.2 to 3.5 (28, 29). The higher prevalence in urban settings is often attributed to sedentary lifestyles, dietary transitions, and greater obesity rates (30). However, once diabetes develops, rural populations may experience a disproportionate burden of complications due to delayed diagnosis, limited access to specialist care, poor medication adherence, and lower awareness of disease management (31). Moreover, glycaemic control is often reported to be poorer among rural residents, even after accounting for socioeconomic factors such as food insecurity. This suboptimal control may contribute to faster disease progression and a higher risk of complications over time (32). A hospital-based study in Bangladesh reported urban prevalence of CAD, stroke, and diabetic foot at 30.5%, 10.1%, and 12.0%, respectively (9), whereas our rural community-based findings were substantially lower. This disparity likely reflects under-diagnosis in rural areas due to limited access to specialised care, diagnostic equipment, and trained professionals. Financial barriers and the absence of health insurance further restrict access to essential tests, while poor health literacy and low awareness delay care-seeking. Moreover, rural health systems have traditionally prioritised infectious diseases over chronic conditions such as diabetes. Consequently, macrovascular complications are probably under-detected and under-reported, meaning the true burden may be far higher than current estimates. These findings are consistent with evidence from South Asia showing that, despite lower diabetes prevalence in rural areas, rural people often present with more advanced disease and poorer outcomes (10, 33). This highlights the urgent need to strengthen screening, early detection, and integrated care in rural settings to reduce disparities in complications between urban and rural populations.

Hypertension and high cholesterol emerged as major predictors of CAD and stroke in this study, reinforcing their central role in atherosclerotic disease development among people with T2DM, particularly with poor glycaemic control. In a large prospective cohort study, hypertension was associated with an 89% higher risk of cerebrovascular disease, while dyslipidaemia and elevated triglycerides increased CAD risk by nearly 50% (34, 35). Similar associations between hypertension, dyslipidaemia, and cardiovascular disease have been reported in large regional and multi-country studies (e.g., INTERHEART; PURE) (36, 37). Accordingly, both European and American guidelines recommend intensive BP control and statin therapy for all people with diabetes, along with glucose-lowering agents that offer cardiovascular benefits, especially for those with existing cardiovascular disease (38, 39). More than three-quarters of this study participants had poor glycaemic control and about two-thirds had hypertension. However, one-fourth of the hypertensive participants were not taking antihypertensive medications as prescribed, with half of them citing financial difficulties, while limited use of lipid-lowering therapy was also observed, both of which may contribute to excess cardiovascular risk. Moreover, although newer classes of glucose-lowering medications glucagon-like peptide-1 (GLP-1) receptor agonists and sodium-glucose cotransporter-2 (SGLT2) inhibitors have been shown to reduce cardiovascular events and mortality through both glycaemic and extra-glycaemic effects and are recommended as first-line therapy for high-risk individuals, their use remains scarce in resource-constrained settings (31). In the current study, none of the participants were using GLP-1 receptor agonists, and a small proportion reported using dipeptidyl peptidase-4 (DPP-4) inhibitors (25%) and SGLT2 inhibitors (4.5%), with most relying on metformin and sulphonylureas.

Smoking showed a strong association with all three macrovascular complications in this study, in line with previous evidence demonstrating smoking as a modifiable risk factor for cardiovascular morbidity and diabetic foot (40). This study also found that higher BMI was associated with CAD. This is consistent with the previous studies reporting that overweight and obese individuals with diabetes have significantly higher risks of CAD compared with those of normal weight (41). Higher BMI increases CAD risk by promoting chronic inflammation, insulin resistance, dyslipidaemia, and hypertension, which are key drivers of atherosclerosis (42). It also contributes to a prothrombotic state and often coexists with other risk factors like poor glycaemic control and metabolic syndrome, compounding cardiovascular risk (43).

The presence of retinopathy across all three complications reflects the interplay between systemic vascular pathology and both microvascular and macrovascular damage (43). Advanced age, longer diabetes duration and onset of diabetes at 45 years or above were also associated with all three complications, aligning with the cumulative effects of prolonged hyperglycaemia and progressive vascular injury (44). These findings are also consistent with the previous studies that have reported similar associations between age-related factors and macrovascular complications (45).

Interestingly, diabetes distress showed a negative association with stroke and diabetic foot, while physical activity was negatively associated with diabetic foot. These findings may reflect reverse causality (46), whereby individuals who develop severe complications receive greater medical attention, counselling, or family support, which can reduce their reported distress despite poorer clinical outcomes. Similarly, patients with diabetic foot may limit activity due to mobility restrictions, leading to paradoxical associations in self-reported physical activity levels. Additionally, potential measurement and reporting bias, particularly in self-reported distress or activity levels, may contribute to these counterintuitive findings. These issues highlight the need for longitudinal designs and objective measures in future research.

In rural Bangladeshi communities, several contextual and structural factors may help explain the prominence of the identified determinants. Limited access to structured diabetes education, inadequate dietary counselling, and strong cultural reliance on carbohydrate-based staple foods contribute to poor glycaemic control and elevated cardiometabolic risk. Sedentary lifestyle patterns, further accentuated by occupational transitions from agriculture to shop-based or domestic work, have increased central adiposity and hypertension risk among adults in these areas. Additionally, healthcare-seeking behaviours are often delayed due to low risk perception, high out-of-pocket treatment costs, and irregular follow-up, collectively limiting continuity of diabetes care. Social norms and caregiving priorities may also commonly place men’s occupational demands and women’s household responsibilities above regular health monitoring, leading to suboptimal adherence to medication and lifestyle recommendations. These contextual constraints provide a plausible explanation for the metabolic and behavioural patterns observed and underscore the importance of culturally adapted intervention strategies.

The findings of this current study have important clinical implications. Integrating cardiovascular risk assessments into routine diabetes care could support earlier detection and intervention in rural communities. Emphasis should be placed on controlling blood pressure and lipids, promoting physical activity, supporting smoking cessation, and addressing psychosocial stressors to reduce macrovascular risk. Improving the availability and affordability of essential medications through subsidies and community-based programmes may further enhance adherence to diabetes management and improve long-term outcomes.

### 4.1 Strengths and limitations

This large-scale, community-based study utilised a representative sample from three rural regions in Bangladesh, strengthening the generalisability of the findings to similar rural populations. The use of a validated electronic questionnaire ensured consistency and reliability in data collection. Moreover, selecting best-performing machine learning algorithms to identify key predictors of macrovascular complications provided a data-driven complement to traditional statistical analyses, enriching the robustness of the results.

Nonetheless, the cross-sectional nature of the study limits the ability to infer temporal or causal relationships between risk factors and health outcomes. While the sample was representative of the selected rural areas, its geographic restriction to three regions may constrain the applicability of findings to the national context. Additionally, reliance on self-reported data for variables such as diet, physical activity, and medication adherence introduces the potential for recall bias, which may affect data accuracy.

## 5 Conclusions

This study highlights a considerable burden of macrovascular complications among adults with T2DM in rural Bangladesh. Key predictors included older age, longer diabetes duration, hypertension, high cholesterol, and smoking. Concurrent microvascular complications were also observed. The use of machine learning enhanced risk factor identification, supporting targeted prevention strategies. Importantly, although rural lifestyles are often perceived as inherently protective, ongoing socio-economic transitions, dietary shifts toward refined carbohydrates, and declining physical activity have increased cardiometabolic risk in these communities, underscoring the need for context-specific lifestyle interventions. For the prevention of complications, our findings underscore the importance of improving HbA1c control, lowering BP, and, where appropriate, initiating statin therapy to lower cholesterol level. Strengthening routine screening, promoting culturally appropriate lifestyle modifications, and implementing integrated chronic care are therefore crucial for reducing complications in rural, low-resource settings.

## Data Availability

The original contributions presented in the study are included in the article/**Supplementary Material**. Further inquiries can be directed to the corresponding author.
