# Supplementary material for: Macrovascular complications in type 2 diabetes: a multiregional study in rural Bangladesh
Source: Front Endocrinol (Lausanne). 2026 Jan 26;17:1724957. doi: 10.3389/fendo.2026.1724957 (PMC12883369; doi:10.3389/fendo.2026.1724957)
Supplement: Supplementary file 1 [file DataSheet1.pdf]

Supplementary Material

Supplementary Figures

A. Coronary artery disease

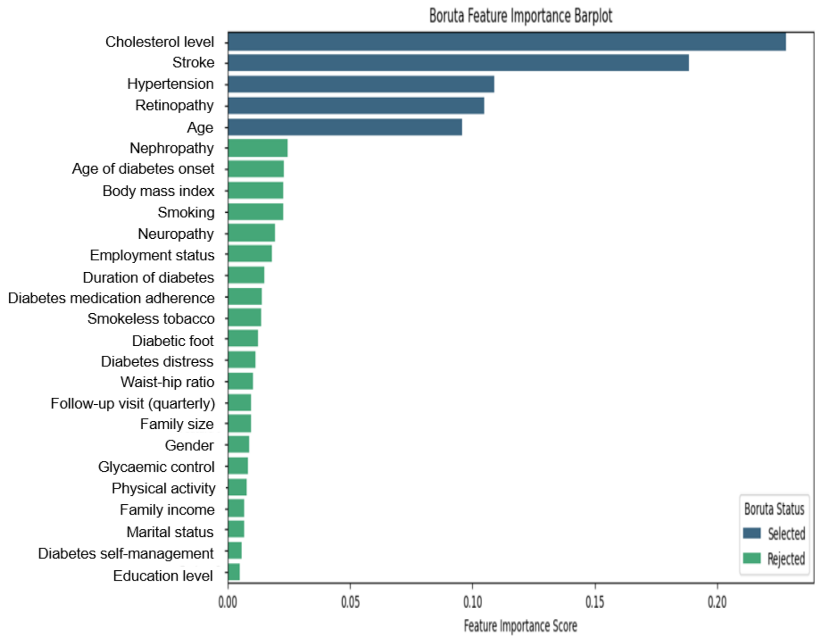

B. Stroke

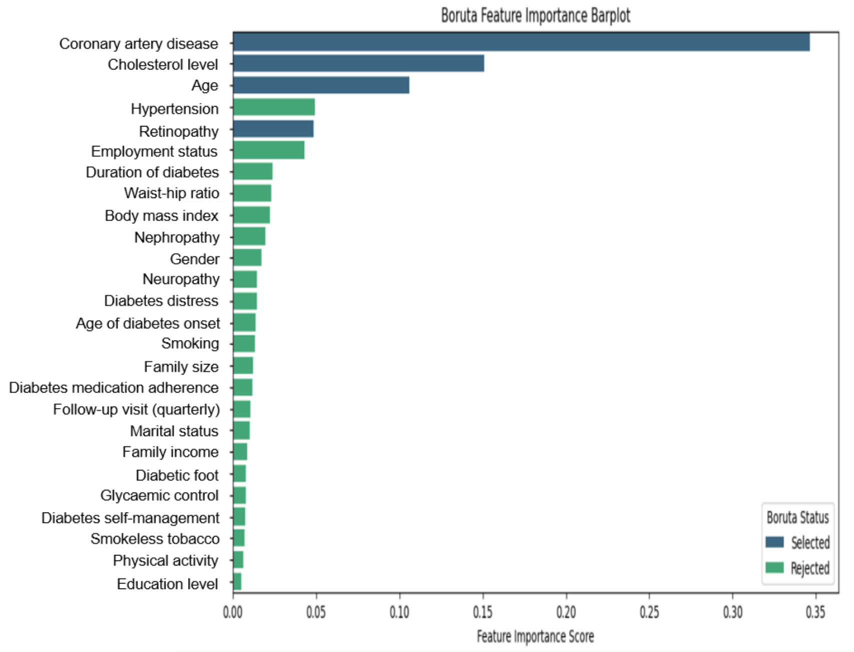

C. Diabetic foot

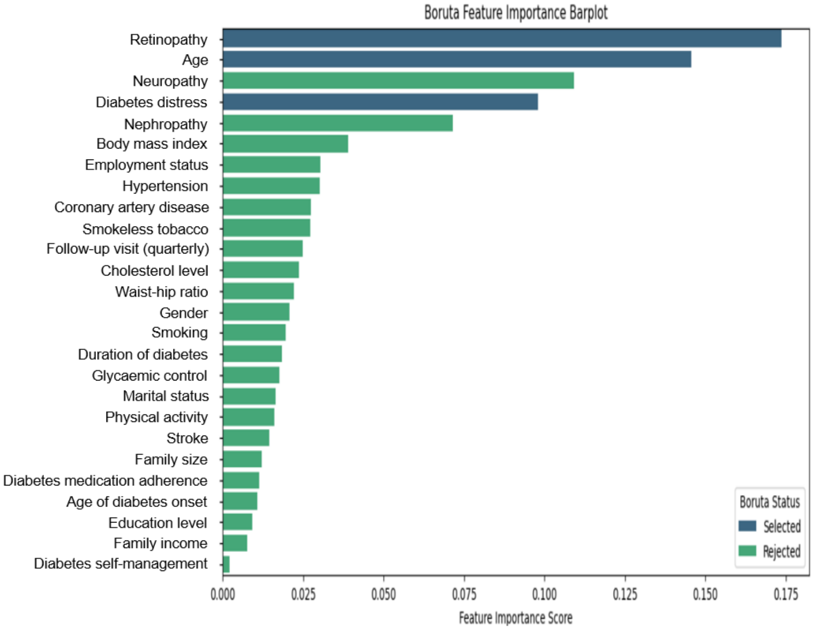

Supplementary Figure 1: Variable selection using the Boruta algorithm. A. Coronary artery disease, B. Stroke and C. Diabetic foot

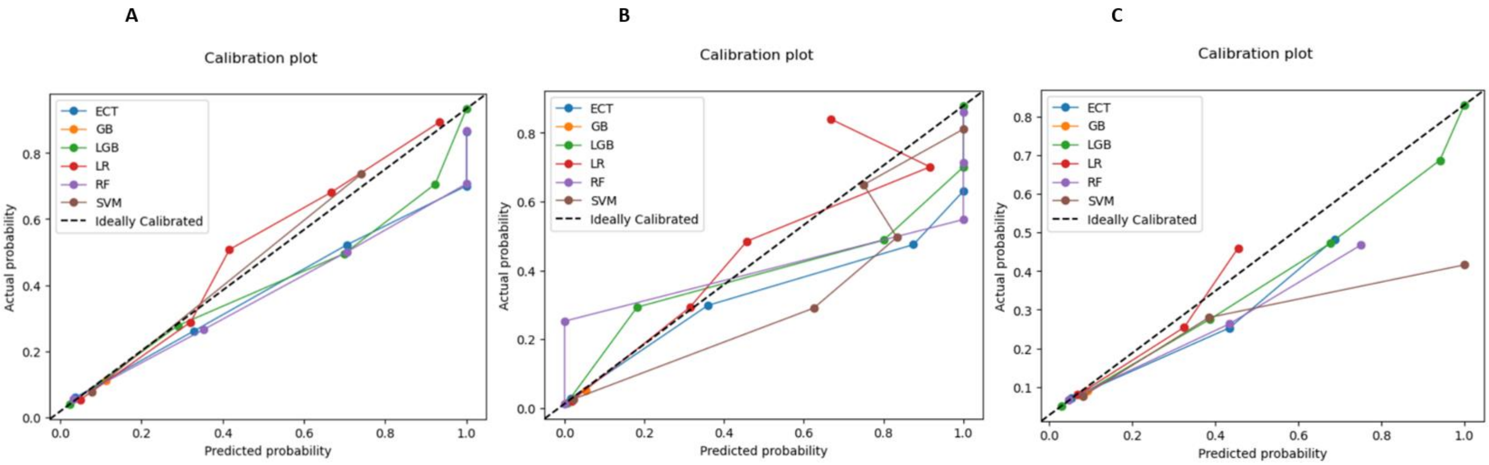

Supplementary Figure 2: Calibration curve for (A) CAD, (B) Stroke and (C) Diabetic foot

Note: ETC, Extra Trees Classifier; GB, Gradient Boosting; LGB, Light Gradient Boosting Machine; LR, Logistic Regression; RF, Random Forest; SVM, Support Vector Machine; CAD, Coronary artery disease

Supplementary Table

Supplementary 1: Multivariable-adjusted associations between macrovascular complications and key risk factors

**Variables**	**CAD***	**Stroke**	**Diabetic foot**
	AOR (95% CI)	AOR (95% CI)	AOR (95% CI)
**Age**	1.00 (0.97–1.04)	1.05 (1.00–1.10)	1.00 (0.98–1.02)
**Employment status**			
Employed (ref)			
Housewives	-	0.92 (0.36–2.35)	-
Retired	-	3.50 (1.07–11.53)	-
**Smoking**			
Never smoked (ref)			
Past/current smoker	2.77 (1.47–5.25)	0.82 (0.29–2.32)	-
**Physical activity**			
Active (ref)			
Inactive	0.59 (0.35–1.01)	-	0.98 (0.58–1.67)
**Diabetes duration group**			
≤5 years (ref)			
6-10 years	0.80 (0.46–1.39)	0.51 (0.22–1.15)	0.97 (0.58–1.62)
>10 years	1.10 (0.54–2.24)	0.53 (0.20–1.43)	0.85 (0.44–1.62)
**Age of DM onset**			
≤45 years (ref)			
>45 years	1.60 (0.77–3.34)	0.40 (0.14–1.09)	-
**Glycaemic control**			
Adequate (ref)			
Inadequate	-	-	1.59 (0.88–2.85)
**Body Mass Index**			
Underweight/Normal (ref)			
Overweight	1.75 (1.01–3.06)	0.43 (0.20–0.94)	0.84 (0.52–1.38)
Obese	1.54 (0.78–3.04)	0.20 (0.10–0.58)	0.48 (0.23–0.98)
**Dyslipidaemia**			
No (ref)			
Yes	4.94 (2.90–8.40)	9.25 (4.06–21.07)	1.68 (0.92–3.07)
**Hypertension**			
No (ref)			
Yes	2.94 (1.48–5.81)	1.96 (0.68–5.69)	1.82 (1.06–3.13)
**Retinopathy**			
No (ref)			
Yes	4.51 (2.60–7.83)	-	3.87 (2.32–6.46)
**Neuropathy**			
No (ref)			
Yes	-	-	3.73 (2.25–6.19)
**CAD**			
No (ref)			
Yes	-	17.05 (8.03–36.19)	-
**Stroke**			
No (ref)			
Yes	14.63 (6.65–32.21)	-	-
**Diabetes distress**			
Little or no distress (ref)			
High distress	-	0.52 (0.24–1.14)	0.26 (0.16–0.42)

*CAD: Coronary artery disease; DM, Diabetes Mellitus

Appendix 1

Questionnaire Development

The structured questionnaire used in this study was developed based on internationally accepted instruments for diabetes and cardiovascular risk assessment, including:

- Global Physical Activity Questionnaire (GPAQ) for physical activity
- American Diabetes Association diabetes monitoring indicators
- Diabetes Distress Scale for psychosocial distress in diabetes
- WHO BMI classification
- Michigan Neuropathy Screening Instrument (MNSI) for neuropathy

Translation, Cultural Adaptation and Validation

The questionnaire was culturally adapted using a rigorous multi-stage translation process to ensure linguistic accuracy and conceptual equivalence. Two bilingual public health experts independently conducted forward translations, which were then reviewed and harmonised by a multidisciplinary panel comprising an endocrinologist, an epidemiologist, and a sociologist. An independent bilingual translator, blinded to the original questionnaire, performed the backward translation to verify consistency. Semantic, idiomatic, experiential, and conceptual equivalence were further assessed through cognitive debriefing interviews with 24 adults with T2DM, confirming clarity, cultural relevance, and comprehension of all items.

Pilot Testing

The refined questionnaire was pilot tested among 24 adults with type 2 diabetes residing in a rural community to assess clarity, comprehension, response burden, and feasibility. Feedback from the pilot led to minor revisions that improved item wording and flow, resulting in a substantial reduction in average completion time from 41 to 28 minutes. These refinements enhanced participant acceptability while maintaining the integrity and completeness of data collection.

Reliability Testing

Internal consistency was calculated using Cronbach’s alpha:

Section / Domain	Cronbach’s α
Physical activity	0.61
Medication adherence	0.63
Diabetes self-management	0.81
Diabetes distress	0.87
SF-12	0.92
ADS	0.79
Neuropathy	0.77
Total instrument score	0.93

Interpretation: $\alpha \geq 0.93$ indicates high internal reliability.

Mode of Administration and Quality Assurance

Data were collected electronically using handheld tablets through the REDCap platform to enhance accuracy and efficiency. Built-in logic checks, skip patterns, and automated completeness alerts were used to minimise entry errors and missing data. Real-time server monitoring allowed continuous oversight of data quality, prevented duplication, and ensured secure and complete data capture throughout the fieldwork period.

Questionnaire Validation and Reliability Summary

Cronbach's alpha was computed for multi-item construct domains only, including physical activity, medication adherence, diabetes self-management and HRQoL sub-scales. Overall the questionnaire demonstrated acceptable reliability ($\alpha > 0.93$). Several components of the questionnaire were adapted from internationally standardised and validated instruments, including GPAQ, DDS-17, MNSI etc. These instruments have been previously validated and widely used in Bangladeshi and South Asian populations, demonstrating acceptable reliability and content validity in similar cultural and epidemiological contexts. As these tools were used without substantive modification, therefore established psychometric properties were retained.

Appendix 2

Questionnaire

For official use only

ID No.				

Preliminary information

a	Date and time of interview	Date:<br><br>Time:
b	Location of data collection	Para:<br>Village:<br>Union:<br>Upazilla:<br>District:
c	Interviewer name	

**Section A1: General and socio-economic information**			
Personal details: *Now I/we would like to collect some personal information about your background*			
Sl no.	Question	Code	Response
A1.1	Participant’s name		
A1.2	Mobile number		
A1.3	Your age (in years)	☐ ☐	
A1.4	Gender (by observation)	0=Male<br>1=Female	
A1.5	What is the highest level of education you have completed?	0 = Never went to school<br>1 = Up to primary school<br>2 = Up to secondary school	

		3 = College completed<br>4 = Graduate/Bachelor<br>5 = Masters or above	
A1.6	What is your marital status?	0 = Never married<br>1 = Currently married<br>2 = Separated/widowed/ divorced	
A1.7	What is your religion?	0 = Islam<br>1 = Hindu<br>2 = Christian<br>3 = Buddhist<br>4 = Others	
A1.8	What is your occupation/working status?	0 = Unemployed……Go to **question A1.9**<br>1 = Service (desk job)<br>2 = Service (field work)<br>3 = Business/Shop<br>4 = Farming<br>5 = Manual/Labour<br>6 = Housewife<br>7 = Retired<br>8 = Others	
A1.9	What is the main reason of your unemployment?	0 = Student<br>1 = Could not find a work<br>2 = Others	
A1.10	Do you have any income?	0 = No…………**go to question A1.13**<br>1 = Yes	
A1.11	What is your income? (monthly average)	…………………………… BDT	
A1.12	Are you the only earning member of the household?	0 = No<br>1 = yes…………**go to question A1.14**	
A1.13	What is your total family income? (monthly average)	………………………….. BDT	
A1.14	How many household members including you live in your house? (eat together)	Number of family members ……………	
A1.15	Participant's average monthly family expenditure	…………………………. BDT	
**Section B2: Life-styles and behavioural information**			
	**Smoking and non-smoking tobacco**		

B2.1	History of smoking (e.g. cigarette, cigars, pipe, bidis)	0 = never smoked …go to QB2.5<br>1 = ex-smoker<br>2 = current smoker QB2.4	
B2.2	How many years have you smoked?	……………..	
B2.3	When was the last time you smoked?	0 = earlier today<br>1 = Not today, but sometime during the past 7 days<br>2 = Not during the past 7 days, but sometimes during the past 30 days<br>3 = Not during the past 30 days, but sometime during the past 6 months<br>4 = Not during the past 6 months, but sometime during the past year<br>5 = 1 to 4 years<br>6 = 5 or more years ago	
B2.4	How many years are/were you smoking?	……………	
B2.5	Have you ever used e-cigarettes, even once or twice?	0=No….skip to QB2.8<br>1=yes	
B2.6	In total, on how many days have you used e-cigarettes in your entire life?	0= 1 day<br>1= 2 to 10 days<br>2= 11 to 20 days<br>3 = 21 to 50 days<br>4 =51 to 51 days<br>5 = Over 100 days	
B2.7	When was the last time you used an e-cigarettes, even one or two times? Choose the first answer that fits.	0 = earlier today<br>1 = Not today, but sometime during the past 7 days<br>2 = Not during the past 7 days, but sometimes during the past 30 days<br>3 = Not during the past 30 days, but sometime during the past 6 months<br>4 = Not during the past 6 months, but sometime during the past year<br>5 = 1 to 4 years<br>6 = 5 or more years ago	
B2.8	History of smokeless tobacco (e.g. zarda, gul, shadapata, khoir)	0 = never used<br>1 = ex-user<br>2 = current user	
	**Physical activity**		
	**Activity at work**		
C3.1	Does your work involve any vigorous-intensity activity that causes large increases in	0 = No, **go to question C3.4**<br>1 = Yes	

	breathing or heart rate like carrying or lifting heavy loads, digging or construction work, working in the agriculture field, fast-swimming for at least 10 minutes continuously?		
C3.2	In a typical week, on how many days do you perform vigorous-intensity activities as part of your work?	Number of days ………………..	
C3.3	How much time do you spend doing these vigorous-intensity activities at work on a typical day? (minutes)	Hours…………… Minutes………………	
C3.4	Does your work involve moderate-intensity activity that causes small increases in breathing or heart rate such as brisk walking, carrying light loads, or cycling for at least 10 minutes continuously?	1 = No, **go to question C3.7**<br>2 = Yes	
C3.5	In a typical week, how many days do you perform moderate-intensity activities as part of your work?	Number of days ……………….	
C3.6	How much time do you spend doing moderate-intensity activities at work on a typical day? (minutes)	Hours …………….. Minutes ……………	
	**Travel to and from places**		
C3.7	Do you walk or use a bicycle (pedal cycle) for at least 10 minutes continuously to get to and from places?	0 = No, **go to question C3.10**<br>1 = Yes	
C3.8	In a typical week, on how many days do you walk or bicycle for at least 10 minutes continuously to get to and from places?	Number of days …………………	
C3.9	How much time do you spend walking or bicycling for travel on a typical day? (minutes)	Hours …………… Minutes ……………..	
	**Recreational activities**		

C3.10	Do you do any vigorous-intensity sports, fitness or recreational (leisure) activities that cause large increases in breathing or heart rate like (running or football) for at least 10 minutes continuously?	0 = No, **go to question to C3.13**<br>1 = Yes	
C3.11	In a typical week, on how many days do you do vigorous-intensity sports, fitness or recreational (leisure) activities?	Number of days …………….	
C3.12	How much time do you spend doing vigorous-intensity sports, fitness or recreational activities on a typical day? (minutes)	Hours ……………. Minutes …………….	
C3.13	Do you do any moderate-intensity sports, fitness or recreational (leisure) activities that causes a small increase in breathing or heart rate such as brisk walking (cycling, swimming, volleyball) for at least 10 minutes continuously?	0 = No, **go to question C3.16**<br>1 = Yes	
C3.14	In a typical week, on how many days do you do moderate-intensity sports, fitness or recreational (leisure) activities?	Number of days …………….	
C3.15	How much time do you spend doing moderate-intensity sports, fitness or recreational (leisure) activities on a typical day? (minutes)	Hours …………… Minutes …………….	
	**Sedentary behaviour**		
	The following question is about sitting or reclining at work, at home, getting to and from places, or with friends including time spent [sitting at a desk, sitting with friends, travelling in car, bus, train, reading, playing cards or watching television], but do not include time spent sleeping.		
C3.16	How much time do you usually spend sitting or	Hours …………… Minutes …………….	

	reclining on a typical day? (hours)		
**Section D4: Diabetes-related general information**			
D4.1	How many years ago were you diagnosed with diabetes?	Years ☐☐ Month ☐☐	
D4.2	How old were you when you were diagnosed with diabetes?	……………	
D4.3	How did you diagnose with diabetes?	0 = during health check-up<br>1 = due to diabetes-related illness<br>2 = due to other illness<br>3 = during pregnancy<br>4 = Others…………………………	
D4.4	In how many pregnancies, it happened?	………..	
D4.5	Where were you diagnosed with diabetes?	0 = Local GP (MBBS)<br>1 = District hospital<br>2 = Upazilla hospital<br>3 = Diabetic clinic/hospital<br>4 = Specialist/endocrinologist<br>5 = Others …………………………	
D4.6	What type of treatment are you taking currently? (may have multiple answers)	0 = diet and exercise<br>1 = one oral medicine<br>2 = more than one oral medicine<br>3 = insulin only<br>4 = oral medicine and insulin<br>5 = Ayurvedic<br>6 = others	
D4.7	List the medications: (brand name)	Name Dose<br>Name Dose<br>Name Dose<br>Name Dose<br>Name Dose	
D4.8	Do you have any side effects of diabetes medication?	0 = No<br>1 = Yes	
D4.9	If yes, which side effect do you have?	0 = Hypoglycemia<br>1 = Gastrointestinal symptoms<br>2 = Weight gain<br>3 = Others, please specify …………………..<br>………………………………………………	
D4.10	Do you have any other family members diagnosed with diabetes?	0 = No ……skip to section E<br>1 = Yes	

D4.11	If yes, could you please mention?	0 = Father<br>1 = Mother<br>2 = Both parents<br>3 = Siblings<br>4 = Others<br>(e.g. Maternal aunt, Paternal grandfather)	
**Section E5: Diabetes-related co-morbidities and complications**			
E5.1	Have you ever been told by a doctor or nurse that you have high blood pressure?	0 = Yes<br>1 = No<br>2 = I don’t know	
E5.2	Have you ever been told by a doctor that you have high lipids or cholesterol?	0 = Yes<br>1 = No<br>2 = I don’t know	
E5.3	Have you ever been told by a doctor that you have heart disease or coronary artery disease (blockage)?	0 = Yes<br>1 = No<br>2 = I don’t know	
E5.4	Have you ever been diagnosed by a doctor with stroke?	0 = Yes<br>1 = No<br>2 = I don’t know	
E5.5	Have you ever been told by a doctor that your diabetes has affected your eyes (retina)?	0 = Yes<br>1 = No<br>2 = I don’t know	
E5.6	Have you ever been told by a doctor that your diabetes has affected your kidney (nephropathy)?	0 = Yes<br>1 = No<br>2 = I don’t know	
E5.7	Do you have pins and needle or numbness in your feet?	0 = Yes<br>1 = No<br>2 = I don’t know	
E5.8	Have ever been told by your doctor that you have had foot problems (injuries, ulcer)?	0 = Yes<br>1 = No<br>2 = I don’t know	
E5.9	If you have one or more of the above diseases/conditions, are you taking prescribed medicine/care (by a medical doctor)?	0 = Yes<br>1 = No<br>2 = I don’t know	
E5.10		If yes, please list the medications<br>………………………………………………<br>………………………………………………<br>………………………………………………	

E5.11	Have you ever been experienced hypoglycaemia (blood glucose <3.9 mmol/L) whilst on treatment in the last 4 weeks? (mild)	0 = No<br>1 = Yes	
E5.12		If yes, number of hypoglycaemic events……	
E5.13	Have you ever been experienced hypoglycaemia (blood glucose <3 mmol/L) whilst on treatment in the last 4 weeks? (moderate)	0 = No<br>1 = Yes	
E5.14		If yes, number of hypoglycaemic events……	
E5.15	Have you ever been experienced severe hypoglycaemia requiring third-party assistance whilst on treatment in the last 4 weeks?<br>(severe)	0 = No<br>1 = Yes	
E5.16		If yes, number of hypoglycaemic events……	
**Section F6: Hypertension related questions**			
F6.1	Do you know or are you aware of your hypertension status?	0 = Yes<br>1 = No ……………skip to F6.14<br>2 = I don't know….. skip to F6.14	
F6.2	How do you know that you have hypertension/blood pressure?	0 = diagnosed by a medical doctor<br>1 = by nurse or from pharmacy<br>2 = through screening program<br>3 = I do not know for sure yet, I am just assuming<br>4 = others	
F6.3	Do you keep record (in a note book/khata, prescription, medical passport) of your blood pressure?	0 = No<br>1 = Yes	
F6.4	When (how long before) were you diagnosed as hypertensive?	0 = within last 5 years<br>1 = more than 5 years ago	
F6.5	Are you aware of any complications of hypertension/high blood pressure (e.g. stroke, heart	0 = No<br>1 = Yes	

	disease, eye problem, kidney problem)?		
F6.6	Are you currently taking any blood pressure medication?	0 = No<br>1 = Yes	
F6.7	How many different medicines a day are you taking?	☐	
F6.8	Are you taking the medicine regularly as advised by your doctor?	0 = No<br>1 = Yes, **go to Question F6.10**	
F6.9	Why don't you take them as advised?	0 = I cannot afford the cost<br>1 = Medicines are not easily available<br>2 = I do not like to take medicines<br>3 = I only take them when I feel sick<br>4 = I do not like the side effects of the medication<br>5 = I prefer alternative medicine<br>6 = I forget to take medicine<br>7 = others	
F6.10	Compared to last 12 months, how is your blood pressure now?	0 = Better<br>1 = Same<br>2 = Worse<br>3 = I don't know	
F6.11	How do you make sure your blood pressure is under control (note: may have multiple answer)?	0 = Take medicines<br>1 = Regular measuring blood pressure<br>2 = Doing regular physical activities<br>3 = Taking healthy food<br>4 = Don't do anything<br>5 = Others	
F6.12	How often do you get your blood pressure checked (note: may have multiple answer)?	0 = Whenever I visit the doctor<br>1 = As advised by the doctor<br>2 = When I do not feel well<br>3 = Others	
F6.13	Do you have any blood relatives (parents, siblings) who has hypertension (high blood pressure)?	0 = No<br>1 = Yes	

F6.14	Why are you not aware of your hypertension status?	0 = I never got measured<br>1 = I don't have symptoms<br>2 = There are no facilities near me to get measured<br>3 = I do not trust the hospitals<br>4 = I am scared I will be diagnosed with it<br>5 = No reason<br>6 = Others	
**Section G7: Diabetes distress**			
G7.1	Feeling that diabetes is taking up too much of my mental and physical energy every day.	1 = not a problem<br>2 = a slight problem<br>3 = a moderate problem<br>4 = somewhat serious problem<br>5 = a serious problem<br>6 = a very serious problem	
G7.2	Feeling overwhelmed by the demands of living with diabetes.	1 = not a problem<br>2 = a slight problem<br>3 = a moderate problem<br>4 = somewhat serious problem<br>5 = a serious problem<br>6 = a very serious problem	
G7.3	Feeling angry, scared, and/or depressed when I think about living with diabetes.	1 = not a problem<br>2 = a slight problem<br>3 = a moderate problem<br>4 = somewhat serious problem<br>5 = a serious problem<br>6 = a very serious problem	
G7.4	Feeling that diabetes controls my life.	1 = not a problem<br>2 = a slight problem<br>3 = a moderate problem<br>4 = somewhat serious problem<br>5 = a serious problem<br>6 = a very serious problem	
G7.5	Feeling that I will end up with serious long-term complications, no matter what I do.	1 = not a problem<br>2 = a slight problem<br>3 = a moderate problem<br>4 = somewhat serious problem<br>5 = a serious problem<br>6 = a very serious problem	

G7.6	Feeling that I am often failing with my diabetes routine.	1 = not a problem<br>2 = a slight problem<br>3 = a moderate problem<br>4 = somewhat serious problem<br>5 = a serious problem<br>6 = a very serious problem	
G7.7	Feeling that I am not sticking closely enough to a good meal plan.	1 = not a problem<br>2 = a slight problem<br>3 = a moderate problem<br>4 = somewhat serious problem<br>5 = a serious problem<br>6 = a very serious problem	
G7.8	Feeling that I am not testing my blood sugars frequently enough.	1 = not a problem<br>2 = a slight problem<br>3 = a moderate problem<br>4 = somewhat serious problem<br>5 = a serious problem<br>6 = a very serious problem	
G7.9	Not feeling motivated to keep up my diabetes self-management.	1 = not a problem<br>2 = a slight problem<br>3 = a moderate problem<br>4 = somewhat serious problem<br>5 = a serious problem<br>6 = a very serious problem	
G7.10	Not feeling confident in my day-to-day ability to manage diabetes.	1 = not a problem<br>2 = a slight problem<br>3 = a moderate problem<br>4 = somewhat serious problem<br>5 = a serious problem<br>6 = a very serious problem	
G7.11	Feeling that my doctor doesn't take my concerns seriously enough.	1 = not a problem<br>2 = a slight problem<br>3 = a moderate problem<br>4 = somewhat serious problem<br>5 = a serious problem<br>6 = a very serious problem	
G7.12	Feeling that my doctor doesn't give me clear enough directions on how to manage my diabetes.	1 = not a problem<br>2 = a slight problem<br>3 = a moderate problem<br>4 = somewhat serious problem<br>5 = a serious problem	

		6 = a very serious problem	
G7.13	Feeling that my doctor doesn't know enough about diabetes and diabetes care.	1 = not a problem<br>2 = a slight problem<br>3 = a moderate problem<br>4 = somewhat serious problem<br>5 = a serious problem<br>6 = a very serious problem	
G7.14	Feeling that I don't have a doctor who I can see regularly enough about my diabetes.	1 = not a problem<br>2 = a slight problem<br>3 = a moderate problem<br>4 = somewhat serious problem<br>5 = a serious problem<br>6 = a very serious problem	
G7.15	Feeling that friends or family don't appreciate how difficult living with diabetes can be.	1 = not a problem<br>2 = a slight problem<br>3 = a moderate problem<br>4 = somewhat serious problem<br>5 = a serious problem<br>6 = a very serious problem	
G7.16	Feeling that friends or family don't give me the emotional support that I would like.	1 = not a problem<br>2 = a slight problem<br>3 = a moderate problem<br>4 = somewhat serious problem<br>5 = a serious problem<br>6 = a very serious problem	
G7.17	Feeling that friends or family are not supportive enough of self-care efforts.	1 = not a problem<br>2 = a slight problem<br>3 = a moderate problem<br>4 = somewhat serious problem<br>5 = a serious problem<br>6 = a very serious problem	
**H8: Michigan Neuropathy Screening Instrument**			
H8.1	Are your legs and/or feet numb?	0 = No<br>1 = Yes	
H8.2	Do you ever have any burning pain in your legs and/or feet?	0 = No<br>1 = Yes	
H8.3	Are your feet too sensitive to touch?	0 = No<br>1 = Yes	
H8.4	Do you get muscle cramps in your legs and/or feet?	0 = No<br>1 = Yes	
H8.5	Do you ever have any prickling feelings in your legs or feet?	0 = No<br>1 = Yes	

H8.6	Does it hurt when the bed covers touch your skin?	0 = No<br>1 = Yes	
H8.7	When you get into the tub or shower, are you able to tell the hot water from the cold water?	0 = No<br>1 = Yes	
H8.8	Have you ever had an open sore on your foot?	0 = No<br>1 = Yes	
H8.9	Has your doctor ever told you that you have diabetic neuropathy?	0 = No<br>1 = Yes	
H8.10	Do you feel weak all over most of the time?	0 = No<br>1 = Yes	
H8.11	Are your symptoms worse at night?	0 = No<br>1 = Yes	
H8.12	Do your legs hurt when you walk?	0 = No<br>1 = Yes	
H8.13	Are you able to sense your feet when you walk?	0 = No<br>1 = Yes	
H8.14	Is the skin on your feet so dry that it cracks open?	0 = No<br>1 = Yes	
H8.15	Have you ever had an amputation?	0 = No<br>1 = Yes	

Section I9: Physical examination of feet

I9.1	Appearance of feet (right): Normal	1 = No<br>2 = Yes	
I9.2	If no, check all that apply:	1 = Deformities<br>2 = Dry skin, callus<br>3 = Infection<br>4 = Fissure	
I9.3	Appearance of feet (left): Normal	1 = No<br>2 = Yes	
I9.4	If no, check all that apply:	1 = Deformities<br>2 = Dry skin, callus<br>3 = Infection<br>4 = Fissure	
I9.5	Ulceration (right foot)	1 = Absent<br>2 = Present	
I9.6	Ulceration (left foot)	1 = Absent<br>2 = Present	
I9.7	Ankle Reflexes (right foot)	1 = Present<br>2 = Present/Reinforcement<br>3 = Absent	

I9.8	Ankle Reflexes (left foot)	1 = Present<br>2 = Present/Reinforcement<br>3 = Absent	
I9.9	Vibration perception at great toe (right)	1 = Present<br>2 = Reduced<br>3 = Absent	
I9.10	Vibration perception at great toe (left)	1 = Present<br>2 = Reduced<br>3 = Absent	
I9.11	10 gm filament (number of applications detected out of 10 applications): right foot	1 = Present (≥8)<br>2 = Reduced (1-7)<br>3 = Absent (0)	
I9.12	10 gm filament (number of applications detected out of 10 applications): left foot	1 = Present (≥8)<br>2 = Reduced (1-7)<br>3 = Absent (0)	

Section J10: Anthropometric measurements and blood pressure

Sl. number	Measurement	Response	Comments
1	Height (cm)		
2	Weight (kg)		
3	Waist circumference (cm)		
4	Hip circumference (cm)		
5	Blood pressure (mm Hg)		

Section K11: Information from patient's guidebook/medical record

Sl. number	Test Name	Result (latest)	Date
1	Fasting blood sugar (FBS)		
2	2 hours after breakfast (AFB)		
3	Random blood sugar (RBS)		
4	Triglycerides (TG)		
5	Low-density lipoprotein (LDL)		
6	High-density lipoprotein (HDL)		
7	Total cholesterol (TC)		
8	Serum creatinine		
9	Urine albumin		
10	Estimated glomerular filtration rate (eGFR)		
11	HbA1c (within 3 months)		
12	HbA1c (done under the project with date)		
